# Vonoprazan-based therapy versus standard regimen for *Helicobacter pylori* infection management in Egypt: an open-label randomized controlled trial

**DOI:** 10.1038/s41598-025-98606-8

**Published:** 2025-05-08

**Authors:** Yumna M. Shekeban, Noha A. Hamdy, Doaa A. Header, Shwikar M. Ahmed, Mai M. Helmy

**Affiliations:** 1 Department of Clinical Pharmacy and Pharmacy Practice, Faculty of Pharmacy, Alexandria University, Alexandria, Egypt; 2 Department of Internal Medicine, Gastroenterology and Endoscopy unit, Faculty of Medicine, Alexandria University, Alexandria, Egypt; 3 Department of Medical Microbiology and immunology, Faculty of Medicine, Alexandria University, Alexandria, Egypt; 4 Department of Pharmacology & Toxicology, Faculty of Pharmacy, Alexandria University, Alexandria, Egypt

**Keywords:** *Helicobacter pylori*, Vonoprazan, Standard triple therapy, Eradication rate, Antimicrobial resistance, Microbiology, Gastroenterology, Health care

## Abstract

As antibiotic resistance continues to rise, the development of novel *H. pylori* treatment regimens, combined with regular assessment of existing treatment regimens, is imperative. The study evaluates the efficacy, safety, and compliance of vonoprazan dual and triple therapy (VDT and VTT) both consisting of vonoprazan 20 mg twice daily and amoxicillin/clavulanate 875 mg/125 three times daily plus clarithromycin 500 mg twice daily added in VTT versus standard triple therapy (STT), in which vonoprazan 20 mg in VTT is replaced by PPI standard or double dose all for 14 days, along with investigating factors influencing compliance, treatment response, and symptoms severity. By per-protocol analysis, the eradication rates of the STT, VDT, and VTT groups were 70%, 76.2%, and 79.2%, respectively (*p* = 0.777) indicating suboptimal efficacy of the three treatment regimens. This necessitates the optimization of dosage and frequency of available treatment regimens as well as the development of new regimens with higher eradication rates. Interestingly, the VDT group demonstrated a better safety profile but with no statistically significant difference in cure rate. No difference in compliance with treatment was found between the groups. Gender, frequency of COVID-19 vaccination, height, and BMI were the only factors assessed influencing infection symptoms severity.

ClinicalTrial.gov ID identifier: NCT05614934, first posted date (07/11/2022).

## Introduction

*Helicobacter pylori (H. pylori)*, a gastric, spiral-shaped gram-negative, microaerophilic pathogen, is one of the most common human pathogens affecting more than half of the world’s population^1^. In Africa, the prevalence of *H. pylori *is reported to be 70.1%, representing one of the highest prevalence regions^2^. While in Egypt, the prevalence is up to 90% as reported by The World Gastroenterology Organization^3^.

*H. pylori *infection is closely associated with a wide spectrum of clinical manifestations including gastrointestinal diseases and extra-gastric manifestations, for instance, chronic gastritis, gastric adenocarcinoma, peptic ulcers, gastric mucosa-associated lymphoid tissue (MALT) lymphoma, and iron deficiency anemia^4^.

*H. pylori *infection represents a major health problem that needs to be treated to prevent serious complications and spread regardless of symptoms^4^. However, eradication of *H. pylori* infection is very challenging due to the high increase in antibiotic resistance rates worldwide, which in turn decreases the effectiveness of currently available eradication regimens and causes the recurrence of *H. pylori* infection.

Primary resistance to clarithromycin, levofloxacin, and metronidazole varies widely regionally, showing different local antimicrobial resistance patterns^3^. A high pooled prevalence of primary and secondary resistance (> 15%) was seen in the majority of WHO areas^5^ and metronidazole resistance was the most prevalent type across all geographical areas^6–8^. Antibiotic resistance patterns in Asia, the US, and Europe were remarkably similar, whereas Africa showed extraordinarily high rates of resistance to all antibiotics^7^. Except for Africa, all regions had low rates of amoxicillin and tetracycline resistance^6^. Limited and controversial data on antibiotic resistance patterns exists in Egypt^9^.

In addition to antimicrobial resistance, poor adherence and irrational use of medical therapy pose a further challenge to managing *H. pylori* infection. Patients’ poor adherence may be attributed to regimens’ adverse effects and regimen complexity.

The appropriate choice of first-line regimen depends on the pattern of local antibiotic resistance known or anticipated^10,11^. Recommended first-line treatments are selected based on clarithromycin resistance. The most commonly recommended triple *H. pylori *regimen composed of amoxicillin, proton pump inhibitor (PPI) and clarithromycin which has been the standard choice for first-line therapy worldwide for many years^12,13^, will provide unacceptably low cure rates in areas of high clarithromycin resistance (≥ 15%)^6^. Despite that, this regimen is still extensively used in Egypt nowadays. Studies have also suggested that eradication rates with other conventional therapies have fallen to unacceptable levels^14^ and that the perfect cure for *H. pylori *infection is still elusive^15^.

Given falling eradication rates for all conventional therapies^14^ in addition to the unavailability of different antimicrobial agents in the Egyptian market such as bismuth salts, rifabutin, furazolidone, and sitafloxacin, there is an urgent need for treatment protocols with a high eradication rate, low antibiotic resistance, and good safety and tolerability. Of these newly proposed regimens are vonoprazan-based regimens. These new regimens should be evaluated for their safety and efficacy in the Egyptian population in order to consider vonoprazan as a global drug.

Vonoprazan is one of the K-competitive acid blockers whose role and value are still emerging. Gastric acid secretion is more consistently and successfully suppressed by K-competitive acid blockers because they are unaffected by the *CYP* *2C19* polymorphism^4^. In addition to their powerful acid-inhibitory effect, vonoprazan-based regimens have been shown to be effective against resistant *H. pylori *strains^16^. Also, vonoprazan-based therapy was proved to be non-inferior to susceptibility-guided PPI-based therapy^17^. Thus, in light of rising antimicrobial resistance, dual therapy using vonoprazan and amoxicillin may result in a breakthrough in the management of *H. pylori*. Through the strong inhibition of gastric acid secretion caused by vonoprazan in addition to the use of just one antibiotic, this regimen may provide a satisfactory rate of *H. pylori *eradication while minimizing antimicrobial resistance at the same time^18^.

Our study aims to assess the safety and efficacy of different *H. pylori* vonoprazan-based regimens (vonoprazan dual therapy, and vonoprazan triple therapy) compared to the commonly used standard triple regimen to eradicate *H. pylori* infection in treatment-naive patients through the determination of each regimen’s eradication rate and reported safety profile. Further objectives of the study include the assessment of patient compliance with eradication regimens as well as the evaluation of the impact of various factors on *H. pylori* infection eradication, patient compliance, and *H. pylori* infection symptoms severity.

## Patients and methods

### Study design and ethical issues

An open-labeled, prospective, three-arm, parallel-group, non-placebo randomized controlled clinical trial was conducted and registered at the Clinical Trials.gov Protocol Registration and Results System (www.clinicaltrials.gov) with the trial registration number NCT05614934.

The study was conducted following the Declaration of Helsinki and the International Conference on Harmonization Guidelines for Good Clinical Practice and was approved by the Ethics Committee of Alexandria University (serial number: 0107175). All study participants were informed about the nature, goal, and potential risks as well as adverse effects of the study and provided their written, informed consent before study entry.

### Study setting

Patients were recruited from the Alexandria Main University outpatient clinics.

### Patients’ eligibility criteria

Patients with dyspeptic symptoms and suspected *H. pylori* infection presented to the outpatients clinics from June 2022 to May 2023 were included for study participation. Patients sensitive to any of the regimens’ components, patients who had received a previous eradication therapy, recent use of antimicrobial agents, proton pump inhibitors, and H_2_ receptor blockers within 1 month, patients with gastric malignancy or those who underwent previous gastric surgery, patients with major concomitant diseases, including psychic disorders and pregnant or lactating women were excluded.

Demographics, full medical and medication history, and data about the symptoms and their severity using the Gastroparesis Cardinal Symptom Index and Modified Gastrointestinal Rating Scale were documented after all participants agreed to take part in this clinical study by signing the written informed consent.

### Sample collection and storage

The diagnosis of *H. pylori* infection was done by stool antigen (HpSA) test using ABON™ One Step *H. pylori* Antigen Test Device (Feces) (ABON Biopharm, Hangzhou) in accordance with manufacturer’s instructions where all stool samples were collected from the study participants in a stool container that is free of media, preservatives, animal serum, or detergents and tested for *H. pylori* infection within 6 h after sample collection. Specimens collected may be stored for 3 days at 2–8 °C if not tested within 6 h. *H. pylori* antigen test device shows a high sensitivity of 100% (95% CI: 96–100%) and specificity of 100% (95% CI: 96–100%) relative to Endoscope-based methods.

Negative subjects were excluded while positive patients were randomly allocated to 3 groups of different *H. pylori* treatment regimens.

### Randomization

All confirmed *H. pylori*-positive patients enrolled in the study were randomly assigned to 3 regimen groups in a 1:1:1 ratio according to a computer-generated random allocation sequence. The randomization used a block size of 6 and no stratification factors.

#### Intervention

Group 1 received a 14-day vonoprazan and high-dose amoxicillin dual therapy, i.e. VDT, (VPZ 20 mg two times a day at 12-hour intervals [BID] and amoxicillin/clavulanate, 875 mg/125 mg three times a day at 8-hour intervals [TID]), group 2 received a 14-day vonoprazan triple therapy, i.e. VTT, (VPZ 20 mg, amoxicillin/clavulanate, 875 mg/125 mg and clarithromycin 500 mg two times a day at 12-hour intervals [BID]) and group 3 received the current standard of care as represented by the standard clarithromycin triple therapy, hereafter STT, (PPI standard or double dose 30 min before meals, clarithromycin 500 mg and amoxicillin/clavulanate, 875 mg/125 mg two times a day at 12-hour intervals [BID]) for 14 days.

### Study outcomes

**Primary outcome**. *H. pylori* status and assessment of regimen effectiveness were performed at least 1 month after treatment completion during a final visit. Successful eradication is defined as a negative *H. pylori *stool antigen test result. Hence, treatment regimen success or failure was detected, and percentage eradication was calculated for each regimen. Following treatment guidelines, PPIs or antimicrobials were not permitted for 2 weeks to 30 days, respectively, before retesting^10^.

**Secondary outcomes.** Medication adherence and adverse events. Patient compliance with different regimens (i.e. medication adherence) was assessed by 2 methods. Firstly, through pill count in medication boxes which is determined during the final visit where the patients were asked to bring all empty medication boxes, and the number of administered and missed pills were counted, a pill intake > 90% is considered good compliance. Secondly providing the patients with a timetable for tracking the intake of medications (patient diaries) where the patients were asked to fill in the table daily during their treatment period and bring the table back with them during the final visit to calculate percentage compliance for each patient. Phone calls were weekly performed to ensure compliance.

For adverse events assessment, patients were reached weekly by phone calls during the treatment period to report any side effect associated with *H. pylori* infection treatment. Any adverse event was considered absent if the subject reported the same complaint at the baseline visit with the same intensity.

The incidence of reported adverse events was checked using a standardized degree of interference with daily activities where mild means not interfering with daily activities, moderate means frequently interfering with daily activities but allowing treatment to be completed, and severe means requiring interruption of treatment.

**Fig. 1:**
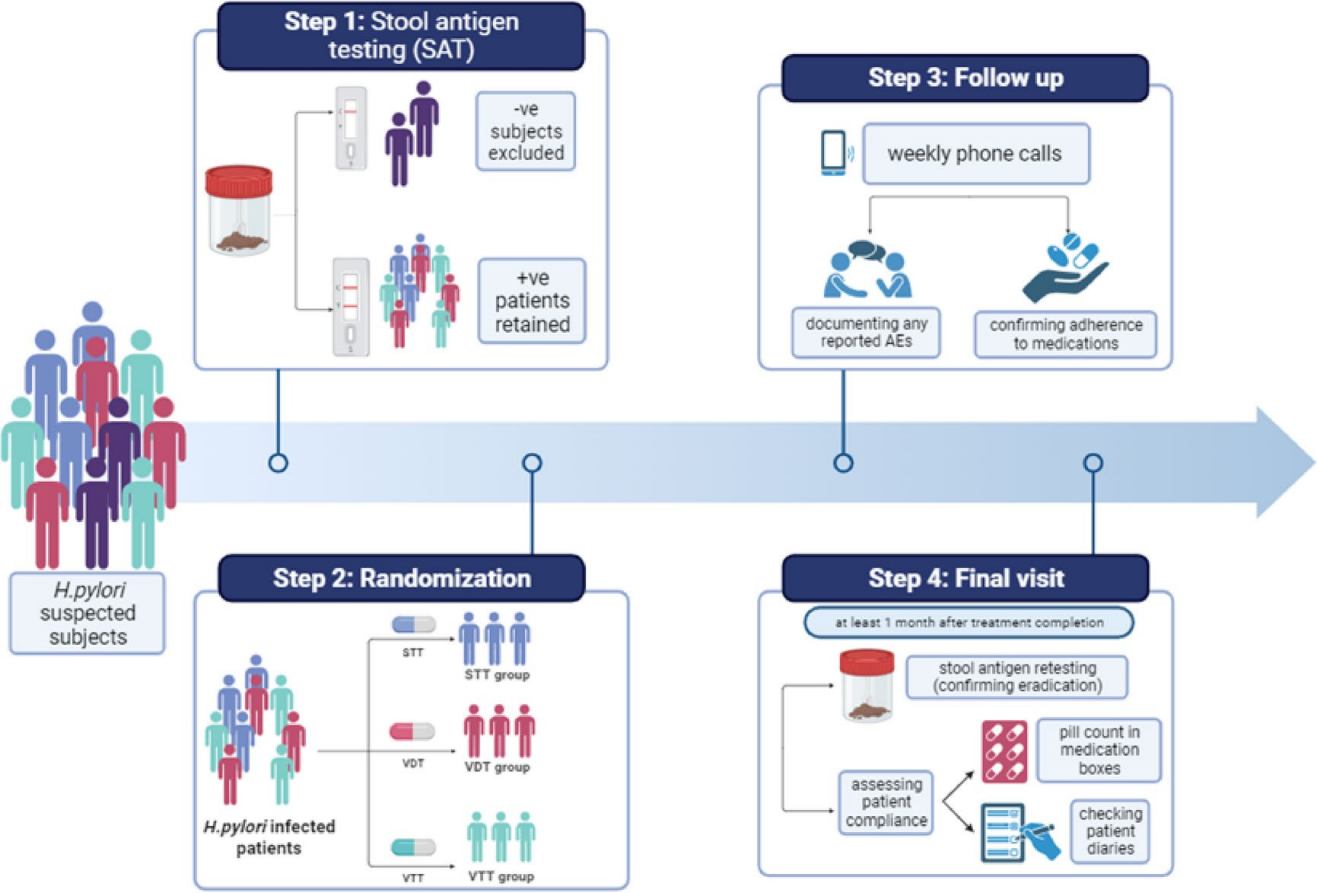
A summary of the study protocol steps. Study includes diagnosis by stool antigen testing followed by subject’s randomization into one of three treatment groups (STT, VDT or VTT) then following up with the patients for documenting compliance and reported adverse events and finally determining each regimen’s eradication rate and percentage compliance. AEs, adverse events; STT, standard triple therapy; VDT, vonoprazan dual therapy; VTT, vonoprazan triple therapy.

Furthermore, the impact of various factors including sex, age, smoking, family history of *H. pylori* infection, previous eradication of *H. pylori* infection, and history of COVID-19 infection and vaccination on *H. pylori* infection eradication, patient compliance, and *H. pylori* infection symptoms severity was determined. Economic impact was also determined by comparing each regimen cost. The study protocol steps are summarized in Fig. 1.

### Sample size

Using open Epi^19^, a sample size of 27 patients per group was calculated considering a statistical power of 80%, a confidence level of 95%, and an *H. pylori *eradication rate of 88.2% in the VDT group, 92.2% in the VTT group, and 60.26% in the STT group^20^.

### Statistical analysis

Intention-to-treat (ITT, including all randomized subjects), modified intention-to-treat (mITT, including subjects who received at least 1 dose and with retesting results), and per-protocol (PP, including subjects who took ≥ 75% of the correct medications and with retesting results) analyses were used to evaluate the primary outcome (eradication rate for each group). The Kolmogorov-Smirnov and Shapiro-Wilk tests were used to test the normal distribution. Consequently, nonparametric tests were used. Differences among the 3 groups were analyzed using the χ2 test, Fisher’s exact test for categorical variables, and the Kruskal-Wallis test for continuous variables. Using Statistical Package for Social Sciences (IBM SPSS^®^ software package version 25.0.) (Armonk, NY: IBM Corp.), data was entered into the computer and analyzed. Qualitative data were described using numbers with percentages in parenthesis while quantitative data were described using median and interquartile range (IQR). The statistical significance of the obtained results was assessed at the 5% level.

Due to the high rate of loss to follow-up in the three treatment groups which led to the presence of missing data about eradication rates and thus may affect the validity of the study results due to bias, a sensitivity analysis test was conducted to check if the study results were robust and reliable. Sensitivity analysis was conducted by comparing the significance resulting from the comparison between the three treatment groups’ eradication rates using ITT analysis while assuming all lost to follow-up patients were not cured (worst case scenario) to the significance while assuming all lost to follow-up patients were cured (best case scenario).

## Results

### Subjects’ enrollment, baseline and clinical characteristics

**Fig. 2:**
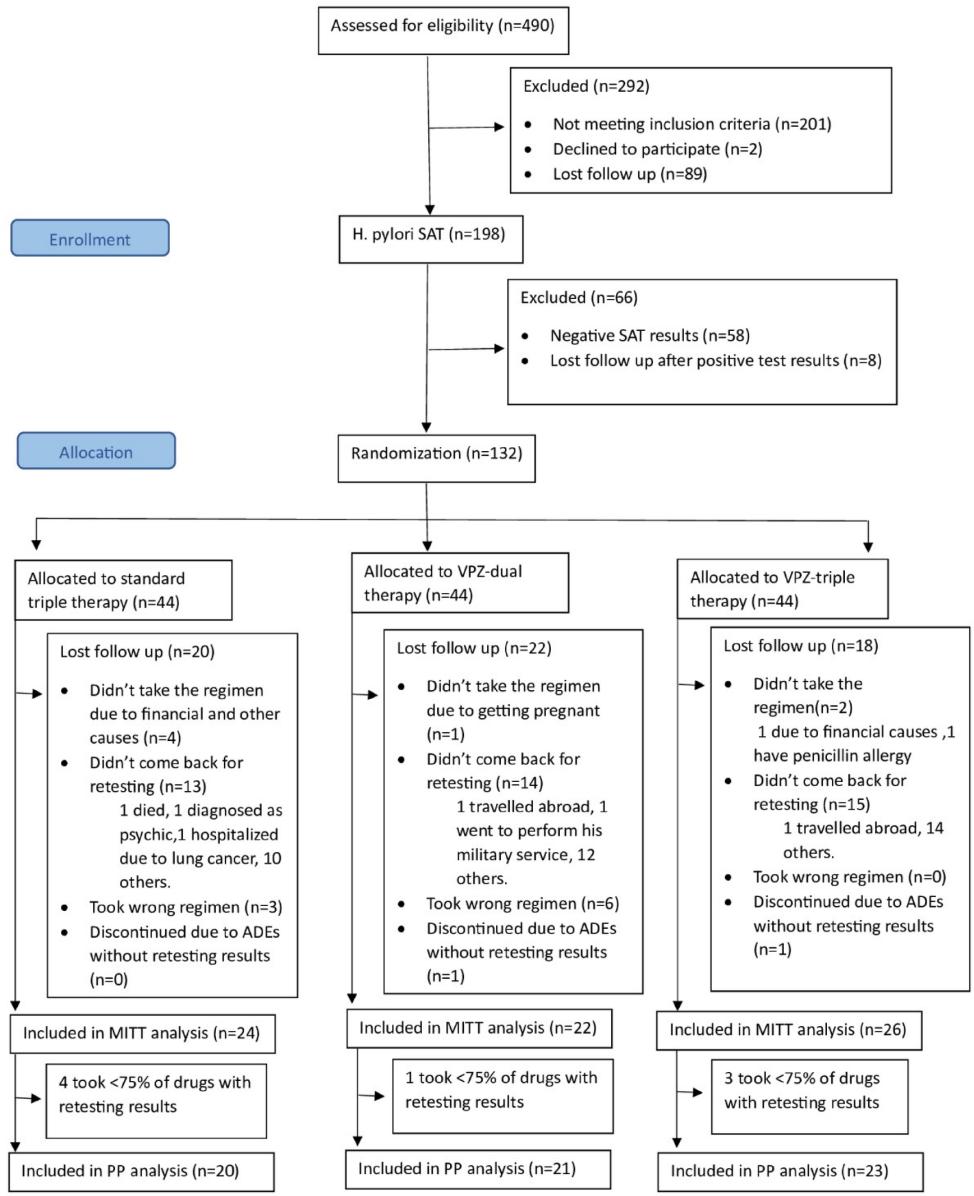
Consort flow diagram of subjects’ enrollment and the progression throughout the trial. ADEs, adverse drug events; mITT, modified intention-to-treat; PP, per protocol; SAT, stool antigen test; VPZ, vonoprazan.

As shown in Fig. 2 490 subjects were screened for study participation and only 198 subjects underwent *H. pylori* stool antigen testing. Finally, a total of 132 subjects were randomly assigned into 3 regimen groups and included in ITT analysis. Among STT, VDT, and VTT groups, 24, 22, and 26 subjects were respectively included in the mITT analysis; 20, 21, and 24 subjects were respectively included in PP analysis.

The ages of subjects in each group were 35.5 (24.5–47) years for the STT group, 31 (21.5–42) years for the VDT, and 37 (28–48) years for the VTT group. Standard PPIs used were either pantoprazole 20 mg or esomeprazole 40 mg. No significant difference was found in the baseline and clinical characteristics among the 3 groups (Table 1) except for the difference between groups in the percentage of subjects having constipation as one *of H. pylori* infection clinical presentations which was higher in the STT group as 52.3% of subjects assigned to STT group had constipation.

The most common reported symptom for *H. pylori* patients was abdominal pain in 90.9% of patients, followed by nausea in 78.8% of patients and bloating in 75% of patients (Table 2).

**Table 1:** Demographic data and baseline characteristics of all randomized subjects indicating no significant difference characteristics among three treatment groups.

Variable	STT	VDT	VTT	*p* value
No. of subjects	44	44	44	-
Age (years)	35.5 (24.5–47)	31 (21.5–42)	37 (28–48)	0.072
Weight (Kg)	73 (65–82)	75 (63–80)	73 (65.5–87)	0.849
Height (m)	1.6 (1.55–1.67)	1.6 (1.55–1.7)	1.64 (1.56–1.72)	0.353
BMI (Kg/m^2^)	26.92 (24.04–32.67)	26.59 (23.04–31.24)	28.19 (23.66–32.24)	0.728
Sex Male Female	12 (27.3%) 32 (72.7%)	13 (29.5%) 31 (70.5%)	13 (29.5%) 31 (70.5%)	0.964
Marital status Single Married Widowed Divorced	14 (31.8%) 24 (54.5%) 4 (9.1%) 2 (1.5%)	16 (36.4%) 27 (64.4%) 1 (2.3%) 0 (0%)	6 (27.3%) 35 (79.5%) 1 (2.3%) 2 (4.5%)	0.054^a^
Smoking Non-smoker Smoker Ex-smoker	33 (75%) 9 (20.5%) 2 (4.5%)	39 (88.6%) 3 (6.8%) 2 (4.5%)	35 (79.5%) 9 (20.5%) 0 (0%)	0.201^a^
Recreational drugs use Yes No	1 (2.3%) 43 (97.7%)	0 (0%) 44 (100%)	2 (4.5%) 42 (95.5%)	0.772^a^
Family history of *H.pylori* infection Yes No	21 (47.7%) 23 (52.3%)	24 (54.5%) 20 (45.5%)	14 (31.8%) 30 (68.2%)	0.089
Surgical history Yes No	16 (36.4%) 28 (63.6%)	20 (45.5%) 24 (54.5%)	24 (54.5%) 20 (45.5%)	0.231
Previous *H.pylori* infection Yes No	3 (6.8%) 41 (93.2%)	9 (20.5%) 35 (79.5%)	8 (18.2%) 36 (81.8%)	0.161
Previous COVID-19 infection Yes No	8 (18.2%) 36 (81.8%)	6 (13.6%) 38 (86.4%)	3 (6.8%) 41 (93.2%)	0.277
COVID-19 vaccination unvaccinated Once Twice Three times	15 (34.1%) 3 (6.8%) 21 (47.7%) 5 (11.4%)	20 (45.5%) 3 (6.8%) 19 (43.2%) 2 (4.5%)	17 (38.6%) 4 (9.1%) 17 (38.6%) 6 (13.6%)	0.782^a^

Data are presented as median and interquartile range for quantitative variables or numbers of subjects with percentages in parenthesis.

^a ^p values calculated using Monte Carlo significance at 99% Confidence Interval as more than 20% of cells have count less than 5.

BMI, body mass index.; STT, standard triple therapy; VDT, vonoprazan dual therapy; VTT, vonoprazan triple therapy.

**Table 2:** Clinical presentation of all randomized subjects showing that abdominal pain, nausea and bloating are the most commonly reported *H. pylori* symptoms.

Clinical presentation	*N*/132	Percentage
Nausea	104	78.8%
Constipation	49	37.1%
Abdominal pain	120	90.9%
Dizziness	13	9.8%
General fatigue	8	6.1%
Halitosis	4	3%
Headache	18	13.6%
Unintentional weight loss	51	38.6%
Loss of appetite	66	50%
Vomiting	38	28.8%
Heartburn	96	72.7%
Diarrhea	58	43.9%
Bloating	99	75%
Frequent burping/belching	46	34.8%
Dyspepsia	96	72.7%
others	9	6.8%

### *H. pylori* eradication rates

As demonstrated in Table 3, the eradication rates of STT, VDT and VTT by intention-to-treat were 38.6%, 38.6% and, 45.5% respectively when assuming that subjects who lost to follow-up weren’t cured (worst case scenario) and 84.1%, 88.6% and, 86.4% respectively, when assuming that subjects who lost to follow up were cured (best case scenario). The results of the sensitivity analysis were consistent as both showed insignificant differences between the treatment groups eradication rates (*p* value = 0.754, 0.824 respectively) thus the results of the sensitivity analysis strengthened the credibility of the conclusion. While the eradication rates of STT, VDT and VTT by modified intention-to-treat were 70.8%, 77.3% and 76.9% respectively and 70%, 76.2% and 79.2% by per-protocol, respectively. Table 3 compared eradication rate for the three treatment groups by PP, mITT and ITT using sensitivity analysis and we found no statistically significant difference in the eradication rates between the three treatment groups with either way of analysis.

**Table 3:** *H. pylori* eradication rates of the three treatment groups showing no statistically significant difference between the three treatment groups.

Analysis	STT	VDT	VTT	*p* value
PP	70% (14/20)	76.2% (16/21)	79.2% (19/24)	0.777
95% CI	45.7–88.1%	52.8–91.8%	57.8–92.9%	
mITT	70.8% (17/24)	77.3% (17/22)	76.9% (20/26)	0.846
95% CI	48.9–87.4%	54.6–92.2%	56.4–91%	
ITT (worst case scenario)	38.6% (17/44)	38.6% (17/44)	45.5% (20/44)	0.754
95% CI	24.4–54.5%	24.4–54.5%	30.4–61.2%	
ITT (best case scenario)	84.1% (37/44)	88.6% (39/44)	86.4% (38/44)	0.824
95% CI	69.9–93.4%	75.4–96.2%	72.6–94.8%	

CI, confidence interval; ITT, intention-to-treat; mITT, modified intention-to-treat; PP, per protocol; STT, standard triple therapy; VDT, vonoprazan dual therapy; VTT, vonoprazan triple therapy.

### Adverse events and compliance

#### a. Adverse events

The most common adverse drug event was taste disturbance in both the STT (73.5% of subjects), and the VTT group (76.3% of subjects), while for the VDT group dizziness was the most common adverse drug event (56.3% of the subjects). While only 40.6% of subjects receiving VDT reported taste disturbance. Taste disturbance in the VDT group was significantly lower than that of the STT group and the VTT group (*p* value = 0.02, 0.007 respectively after Bonferroni correction). Otherwise, the incidence of adverse events wasn’t significantly different between groups.

The overall number of adverse drug events in the STT group is 135 events, 109 events in the VDT group and 157 events in the VTT group. The VDT group showed the least number of events per subject among the three treatment groups.15, 3 and 7 severe adverse drug events caused the subjects to discontinue the medications for a while, this was reported by 3, 2 and 3 subjects receiving STT, VDT and VTT respectively. One subject in VDT group and another in VTT group discontinued the medications completely due to treatment-associated adverse drug events. No subjects were hospitalized because of adverse events. (Table 4)

**Table 4:** Adverse events and compliance of the three treatment groups.

Adverse event	STT (*n* = 34)	VDT (*n* = 32)	VTT (*n* = 38)	*p* value
Diarrhea	14 (41.2%)	12 (37.5%)	23 (60.5%)	0.11
Mild	7 (20.6%)	3 (9.4%)	12 (31.6%)	0.048*^a^
Moderate	5 (14.7%)	9 (28.1%)	11 (28.9%)	
Severe	2 (5.9%)	0 (0%)	0 (0%)	
Taste disturbance	25 (73.5%)	13 (40.6%)	29 (76.3%)	0.003*
Mild	8 (23.5%)	12 (37.5%)	16 (42.1%)	0.001* ^a^
Moderate	14 (41.2%)	1 (3.1%)	12 (31.6%)	
Severe	3 (8.8%)	0 (0%)	1 (2.6%)	
Bloating	7 (20.6%)	7 (21.9%)	8 (21.1%)	0.992
Mild	6 (17.6%)	4 (12.5%)	4 (10.5%)	0.534 ^a^
Moderate	1 (2.9%)	3 (9.4%)	2 (5.3%)	
Severe	0 (0%)	0 (0%)	2 (5.3%)	
Loss of appetite	11 (32.4%)	8 (25%)	11 (28.9%)	0.805
Mild	4 (11.8%)	7 (21.9%)	7 (18.4%)	0.268 ^a^
Moderate	5 (14.7%)	1 (3.1%)	4 (10.5%)	
Severe	2 (5.9%)	0 (0%)	0 (0%)	
Nausea	11 (32.4%)	5 (15.6%)	15 (39.5%)	0.087
Mild	6 (17.6%)	4 (12.5%)	11 (28.9%)	0.346 ^a^
Moderate	3 (8.8%)	1 (3.1%)	3 (7.9%)	
Severe	2 (5.9%)	0 (0%)	1 (2.6%)	
Vomiting	2 (5.9%)	1 (3.1%)	5 (13.2%)	0.283 ^a^
Mild	0 (0%)	1 (3.1%)	3 (7.9%)	0.692 ^a^
Moderate	1 (2.9%)	0 (0%)	1 (2.6%)	
Severe	1 (2.9%)	0 (0%)	1 (2.6%)	
Abdominal pain	13 (38.2%)	14 (43.8%)	17 (44.7%)	0.839
Mild	5 (14.7%)	6 (18.8%)	9 (23.7%)	0.783 ^a^
Moderate	6 (17.6%)	8 (25%)	7 (18.4%)	
Severe	2 (5.9%)	0 (0%)	1 (2.6%)	
Constipation	1 (2.9%)	9 (28.1%)	6 (15.8%)	0.018
Mild	1 (2.9%)	5 (15.6%)	6 (15.8%)	0.006* ^a^
Moderate	0 (0%)	4 (12.5%)	0 (0%)	
Severe	0 (0%)	0 (0%)	0 (0%)	
Skin rash	6 (17.6%)	3 (9.4%)	3 (7.9%)	0.404 ^a^
Mild	5 (14.7%)	2 (6.3%)	2 (5.3%)	0.769 ^a^
Moderate	1 (2.9%)	1 (3.1%)	1 (2.6%)	
Severe	0 (0%)	0 (0%)	0 (0%)	
Dizziness	17 (50%)	18 (56.3%)	15 (39.5%)	0.362
Mild	8 (23.5%)	9 (28.1%0	11 (28.9%)	0.547 ^a^
Moderate	7 (20.6%)	7 (21.9%)	4 (10.5%)	
Severe	2 (5.9%)	2 (6.3%)	0 (0%)	
Headache	14 (41.2%)	7 (21.9%)	11 (28.9%)	0.226
Mild	8 (23.5%)	2 (6.3%)	8 (21.1%)	0.450 ^a^
Moderate	5 (14.7%)	4 (12.5%)	2 (5.3%)	
Severe	1 (2.9%)	1 (3.1%)	1 (2.6%)	
Hypersomnia	5 (14.7%)	1 (3.1%)	6 (15.8%)	0.199 ^a^
General fatigue	3 (8.8%)	6 (18.8%)	5 (13.2%)	0.515 ^a^
Xerostomia	1(2.9%)	1(3.1%)	1(2.6%)	1 ^a^
Others	5 (14.7%)	4 (12.5%)	2 (5.3%)	0.406
Total				
Events	135	109	157	-
N^0^ of events/subject	3.9:1	3.4:1	4.1:1	-
Subjects	31 (91.2%)	30 (93.8%)	36 (94.7%)	0.889 ^a^
Mild events	58	55	89	-
Moderate events	48	39	47	-
Severe				
Events	15	3	7	-
subjects	3 (8.8%)	2 (6.3%)	3 (7.9%)	1 ^a^
Non graded events	14	12	14	-
Discontinued medication due to side effects	0/44 (0%)	1/44 (2.27%)	1/44 (2.27%)	0.988

^a^ p values calculated using Monte Carlo significance at 99% Confidence Interval as more than 20% of cells have count less than 5.

Data are expressed as number of subjects suffering from an adverse event and percentage in parenthesis or as number of adverse events.

#### b. Compliance

Data on compliance with the prescribed medications were available for 96 subjects: 32, 31 and 33 subjects in STT, VDT and VTT groups respectively. No significant difference was detected in the patient adherence to medications among the three treatment groups as the median was 100% (range 29.76 − 100%) in the STT group, 97.14% (range 42.85 − 100%) in the VDT group and 100% (range 35.71 − 100%) in the VTT group (*p* value = 0.725).

**Fig. 3:**
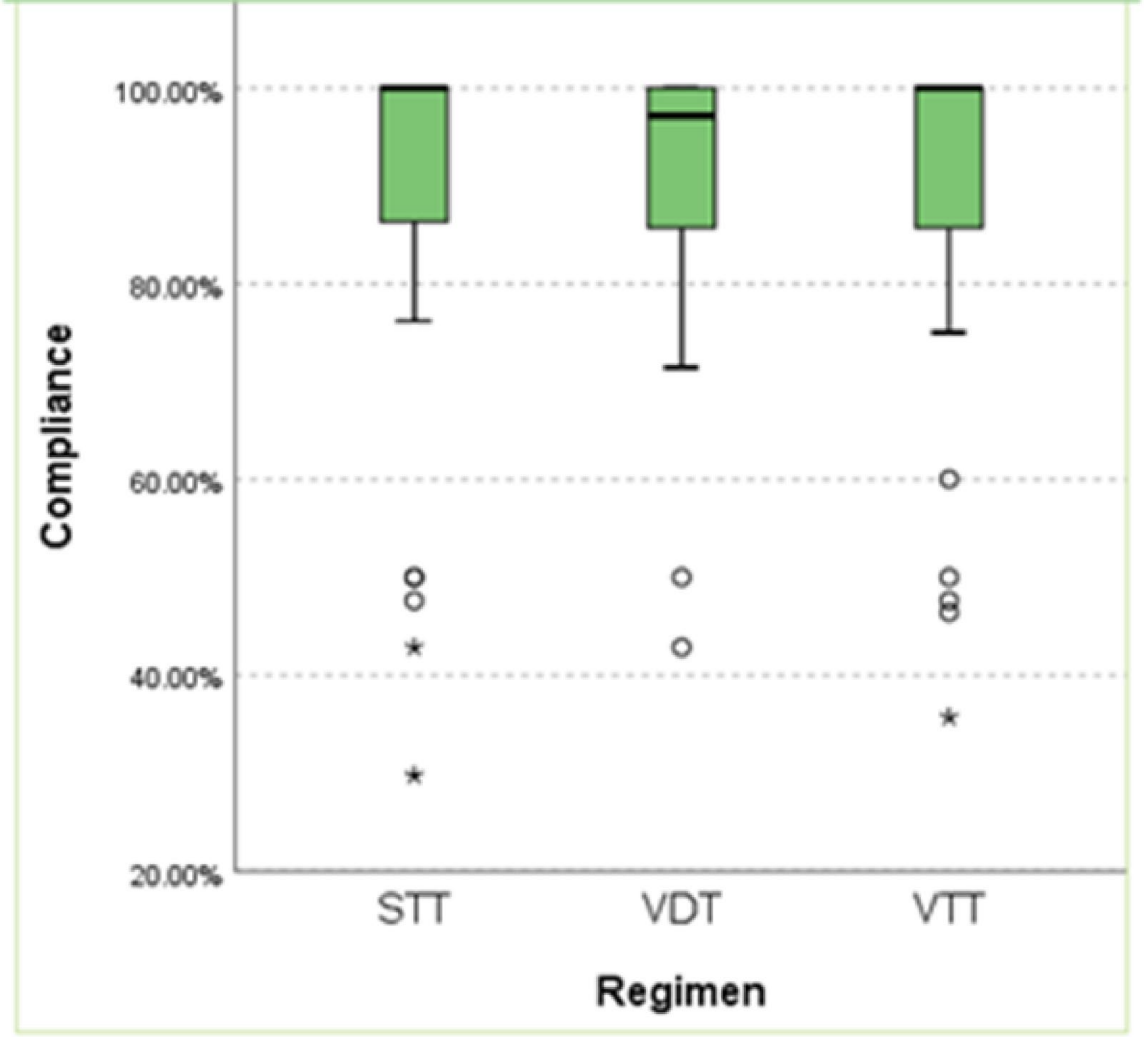
The difference in subjects’ compliance between the three treatment groups, No significant difference was detected in the patient adherence to medications among the three treatment groups. STT, standard triple therapy; VDT, vonoprazan dual therapy; VTT, vonoprazan triple therapy.

Overall, 71.87% (23/32), 70.96% (22/31) and 69.69% (23/33) of subjects taking STT, VDT and VTT regimens, respectively, completed more than 90% of their medications which is considered good compliance. Figure 3.

### Univariate analysis of the demographic and clinical factors influencing *H. pylori* eradication rate, *H. pylori* infection symptoms severity and patient compliance to medications

#### a. Determining factors affecting treatment eradication rate

None of the evaluated factors including sex, age, symptom severity score, smoking, post-treatment use of antibiotics, previous eradication of *H. pylori* infection and patient compliance with treatment regimens were found to affect the patient’s response to treatment.

#### b. Determining factors affecting *H. pylori* infection symptoms severity

*H. pylori* symptom severity expressed by the GSRS symptom severity score was found to be affected by sex. Females reported more severe symptoms than males as the median score for males and females was 10 (6–14) and 15 (10–18) respectively (*p* = 0.001). Moreover, the number of the COVID-19 vaccine doses administered has significantly affected the severity of *H. pylori* infection symptoms where the median GSRS severity score was 11.5 (6–16), 19 (16–20), 13 (10–17) and 16 (14–21) for the unvaccinated group, group of subjects vaccinated once, twice and three times respectively. (*p* value = 0.005) (Table 5).

**Fig. 4:**
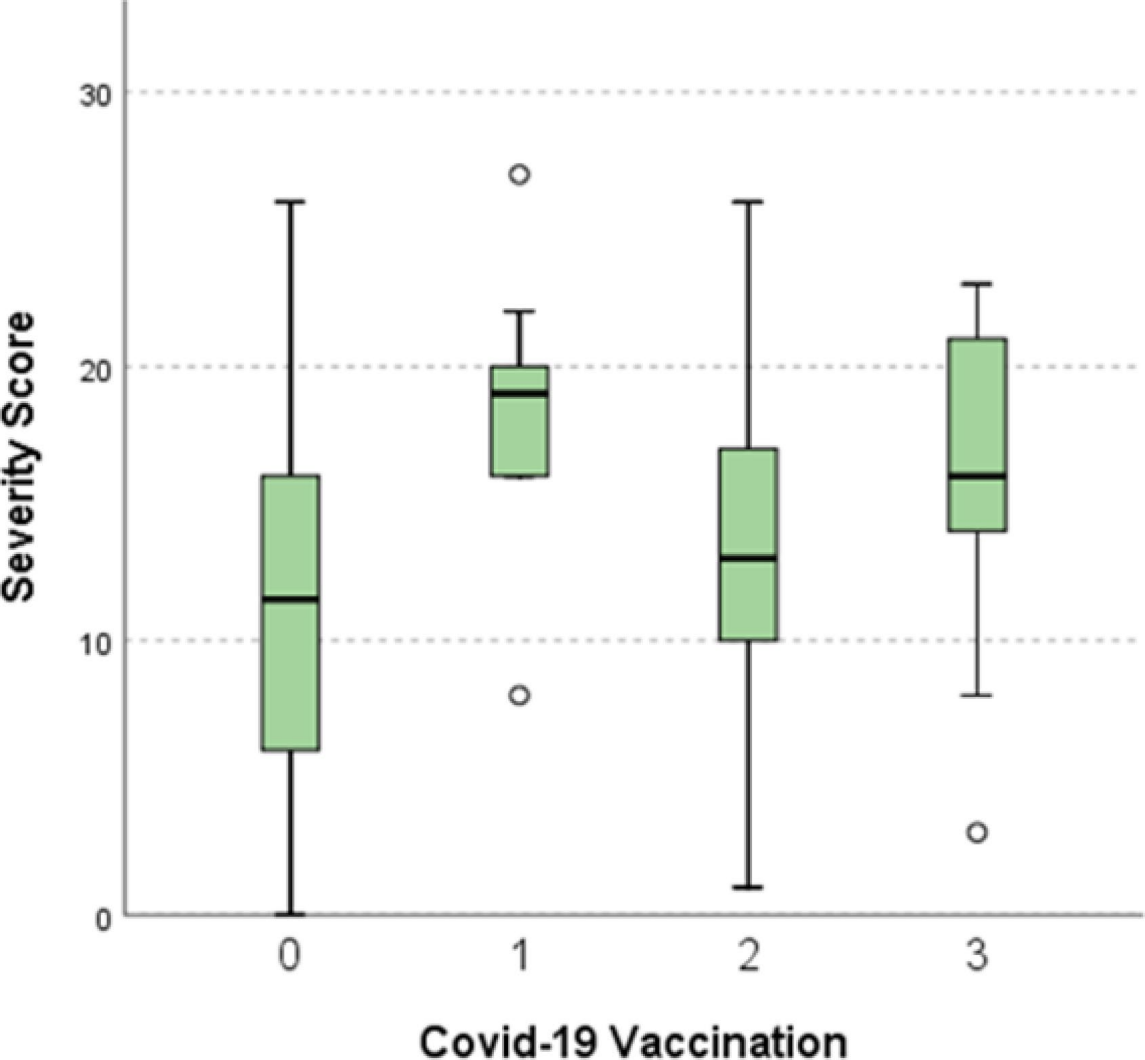
The difference in GSRS symptom severity score according to numbers of COVID-19 vaccine doses administered. A significant difference between severity scores of the unvaccinated group and the group vaccinated three times, the unvaccinated group and the group vaccinated once and between the group vaccinated once and the group vaccinated twice. 0, unvaccinated; 1, vaccinated once; 2, vaccinated twice; 3, vaccinated three times.

Pairwise comparisons of GSRS severity score revealed a significant difference between severity scores of the unvaccinated group and the group vaccinated three times (*p* value = 0.015), the unvaccinated group and the group vaccinated once (*p* value = 0.003) and between the group vaccinated once and the group vaccinated twice (*p* value = 0.023) (Fig. 4).

**Table 5:** The association between different variables and GSRS severity score.

Factor	GSRS severity score	*p* value
Sex Male Female	10 (6–14) 15 (10–18)	0.001*
Marital status Single Married Widowed Divorced	12.5 (8–16.5) 14 (10–17) 11 (3–16) 16.5 (8.5–18.5)	0.508
Smoking Non-smoker Smoker Ex-smoker	14 (9–17) 13 (8–17) 12.5 (8.5–13.5)	0.666
Recreational drugs use Yes No	19 (13.5–19.5) 14 (9–17)	0.428
Family history of *H.pylori* infection Yes No	15 (10–17) 13 (8–17)	0.324
Surgical history Yes No	14 (6.5–17) 13.5 (10–17.5)	0.441
Previous *H.pylori* infection Yes No	13.5 (8.5–17) 14 (9–17)	0.98
Previous COVID-19 infection Yes No	15 (11–18) 14 (8.5–17)	0.382
COVID-19 vaccination unvaccinated Once Twice Three times	11.5 (6–16) 19 (16–20) 13 (10–17) 16 (14–21)	0.005*

As reported in Table 6, the subject’s height was found to negatively correlate with the GSRS symptom severity score. The lower the height, the higher GSRS symptom severity score. Furthermore, the correlation between BMI and GSRS symptom severity score was significant. However, both height and BMI showed a weak correlation (*p* = 0.016, 0.003 respectively).

**Table 6:** Correlation between different variables and GSRS severity score.

Factor	GSRS severity score	
	Spearman correlation coefficient (*r*_s_)	Significance
Age	0.074	0.399
Weight	0.143	0.103
Height	−0.210	0.016*
BMI	0.260	0.003*

BMI, body mass index; GSRS, Modified Gastrointestinal Symptom Rating Scale.

Correlation is significant at the 0.05 level (2-tailed).

#### c. Determining factors affecting patient compliance with medications

None of the factors was found to affect patient compliance with treatment regimens significantly. (Tables 7 and 8)

**Table 7:** Association between different variables and patient compliance.

Factor	Compliance	*p* value
Sex Male Female	98.8% (91.42 − 100%) 100% (83.92 − 100%)	0.993
Marital status Single Married Widowed	98.57% (88.09 − 100%) 100% (85.71 − 100%) 100% (98.83 − 100%)	0.651
Smoking Non-smoker Smoker Ex-smoker	100% (85.71 − 100%) 100% (94.87 − 100%) 95.71% (94.87 − 97.25%)	0.432
Recreational drugs use Yes No	100% (85.71 − 100%) 100% (73.80 − 100%)	0.953
Family history of *H.pylori* infection Yes No	100% (91.42 − 100%) 100% (82.14 − 100%)	0.323
Surgical history Yes No	100% (89.28 − 100%) 100% (83.92 − 100%)	0.441
Previous *H.pylori* infection Yes No	100% (88.57–100%) 100% (85.71 − 100%)	0.746
Previous COVID-19 infection Yes No	98.57% (85.71 − 100%) 100% (85.71 − 100%)	0.791
COVID-19 vaccination unvaccinated Once Twice Three times	98.20% (82.14 − 100%) 94.64% (68.44 − 100%) 100% (92.13 − 100%) 100% (88.68 − 100%)	0.691

**Table 8:** Correlation between different variables and percentage compliance to treatment regimens.

Factor	% compliance	
	Spearman correlation coefficient (*r*_s_)	Significance
Age	0.124	0.230
Weight	0.106	0.305
Height	0.090	0.383
BMI	0.011	0.916
GSRS severity score	−0.128	0.214

BMI, body mass index; GSRS, Modified Gastrointestinal Symptom Rating Scale.

Correlation is significant at the 0.05 level (2-tailed).

### Economic impact

Based on the Egyptian market price (Egyptian pound (EGP)), Vonoprazan’s average price was slightly higher than PPI. However, the overall average cost of the VDT regimen (560 EGP) was less than both the VTT (655.2 EGP) and STT regimens (621.6 EGP).

## Discussion

Despite the availability of studies comparing vonoprazan-based regimens with other standard-of-care regimens for the treatment of *H. pylori* infection favoring the use of vonoprazan-based regimens, their use as a first-line therapy is still not recommended in recent *H. pylori *treatment guidelines, which indicate that vonoprazan’s role and value are still being explored^21^. The reason for this is that there are no enough studies available, and the majority of studies were limited to using 7-day regimens and to countries in the Far East and Japan, and no evidence from other countries with fewer resources or longer duration regimens. Thus, the results of these studies cannot be generalized worldwide coupled with a strong demand for high-quality data from countries where *H. pylori*-related diseases are most prevalent such as Egypt.

In agreement with the results of previous studies^4,22–24^, our study has confirmed that the efficacy of the standard clarithromycin triple therapy has fallen to suboptimal levels which is considered to be unacceptable or F category according to the report card format suggested by Graham et al. to evaluate the outcome of treatment regimens for *H. pylori *infection^25^and that the standard clarithromycin triple therapy use should only be restricted to areas with well documented low clarithromycin resistance rates of less than 15% as recommended by the Maastricht V/Florence Consensus Report and ACG clinical guidelines^10,11^.

Almost all studies conducted in Japan reported that vonoprazan-based regimens achieved higher eradication rates than standard clarithromycin triple therapy as a first-line treatment for *H.pylori*^26–34^. A summary of some of these studies is provided in Table 9.

**Table 9:** A summary of Japanese studies assessing the efficacy of vonoprazan-based regimens versus standard clarithromycin triple therapy.

Reference	Date	Dosage of vonoprazan/PPIs	Dosage of antibiotics	Duration (days)	Eradication rates (vonoprazan/PPIs)	*p* value
^33^	2019	vonoprazan 20 mg bid /lansoprazole 30 mg or rabeprazole 10 mg bid	clarithromycin 400 mg bid and amoxicillin 750 mg bid	7	90.2% /71.9%	*p* < 0.00001
^31^	2018	vonoprazan 20 mg bid /lansoprazole 30 mg bid	clarithromycin 200 or 400 mg bid and amoxicillin 750 mg bid	7	91.0% /84.7%	*p* = 0.030
^27^	2017	vonoprazan 20 mg bid /lansoprazole 30 mg or rabeprazole 20 mg bid	clarithromycin 200 or 400 mg bid and amoxicillin 750 mg bid	7	95.8%/69.6% (ITT analysis); 95.7%/71.4% (PP analysis)	*p* = 0.00003 by ITT analysis *P* = 0.0002 by PP analysis
^26^	2016	vonoprazan 20 mg bid /lansoprazole 30 mg bid	clarithromycin 200 or 400 mg bid and amoxicillin 750 mg bid	7	92.6% /75.9%	*p* < 0.0001
^34^	2016	vonoprazan 20 mg bid /esomeprazole 20 mg bid	clarithromycin 200 mg bid and amoxicillin 750 mg bid	7	84.6%/79.1% (by ITT analysis); 86.3%/79.9% (by PP analysis)	*p* = 0.021 by ITT analysis *p* = 0.019 by PP analysis
^29^	2016	vonoprazan 20 mg bid /lansoprazole 30 mg or rabeprazole 10 mg or esomeprazole 20 mg or omeprazole 20 mg	clarithromycin 200 or 400 mg bid and amoxicillin 750 mg bid	7	87.2% /72.4%	*p* < 0.01
^30^	2016	vonoprazan 20 mg bid /lansoprazole 30 mg or rabeprazole 20 mg	clarithromycin 200 mg bid and amoxicillin 750 mg bid	7	89.1%/70.9% (by ITT analysis); 91.2/71.7% (by PP analysis)	*p* < 0.001 by ITT and PP analysis

Bid, twice daily; ITT, intention-to-treat; PP, per protocol; PPIs, proton pump inhibitors.

In the current study, vonoprazan-based regimens demonstrated higher eradication rates than standard triple therapy, VTT showed higher eradication rates by both ITT and PP analyses and VDT demonstrated higher rates by MITT analysis. However, this difference in eradication rates between the three treatment groups using either way of analysis was not statistically significant. Similarly, Shinozaki et al. found no significant differences between vonoprazan-based triple therapy and esomeprazole-based triple therapy^35^. The reason behind the slightly higher efficacy of vonoprazan-based regimens is that potassium competitive acid blockers provide faster, more potent, and more prolonged gastric acid inhibition than PPIs^3,36^. This in turn enhances the efficacy of protein-targeted antibiotics such as clarithromycin and amoxicillin through different mechanisms: firstly, sustained elevated intragastric pH allows for *H. pylori *multiplication and thus increases the sensitivity of these antibiotics that work effectively during the bacterial growth phase^32,37^. Secondly, both amoxicillin and clarithromycin are acid-sensitive antibiotics. As a result, adequate inhibition of gastric acid secretion and maintaining intragastric pH above 5 for a longer time increases their stability and bioavailability^23,34,38–40^. Thirdly, amoxicillin exists as a zwitter ion between pH 3 and 6. Its high lipophilicity and net charge of 0 allow for better diffusion through biological membranes, which enhances its activity^41^. In addition to being pH-dependent, amoxicillin is also a time-dependent antibiotic^42^which means that it is more effective when high intragastric pH time is prolonged which is the case while using vonoprazan. Furthermore, PPIs are primarily metabolized by CYP 2C19 and the CYP 2C19 polymorphism may lead to reduced gastric acid inhibition in extensive metabolizers, leading to treatment failure^43–45^. Vonoprazan, on the other hand, is mainly metabolized by CYP 3A4/5^46^and exhibits consistent gastric acid inhibition with no individual variation^32^.

According to the study findings, VTT achieved a higher eradication rate than VDT which supports previous studies conducted in Japan, the United States, and Europe^23,47^. In contrast, Furuta et al. achieved a higher eradication rate by VDT than by VTT although these differences in the eradication rates were not significant^36^. The slightly higher efficacy of VTT could be explained by the synergistic effect of vonoprazan and clarithromycin both of which are mainly metabolized by CYP 3A4 and are potential inhibitors of CYP 3A4 when used concomitantly. As a result, vonoprazan and clarithromycin may impede each other’s metabolism, raising their plasma concentrations and enhancing the overall effectiveness of the regimen^48^. Nevertheless, this regimen consists of two antimicrobial agents, which means that it will contribute more to the growing antimicrobial resistance patterns. Furthermore, one of these antibiotics is clarithromycin which is a potent inhibitor of both CYP 3A4 and P-glycoprotein^49,50^. As a result, it alters plasma concentrations of P-glycoprotein and CYP 3A4 substrates potentially leading to increased toxicity. Therefore, patients receiving VTT in conjunction with multiple medications should take extra precautions. Clarithromycin is also associated with an increase in the risk of ventricular arrhythmia due to QT interval prolongation^51^. Consequently, clarithromycin entails an additional risk of drug-drug interactions and side effects to the regimen. On the other hand, VDT is a single antimicrobial regimen with comparable eradication rates to VTT as well as STT, which in accordance with antibiotic stewardship principles reduces unnecessary use of antibiotics and provides comparable treatment outcomes with less contribution to the development of secondary antimicrobial resistance.

Notably, the eradication rates attained in this study are suboptimal and not as high as those reported in previous Japanese studies and are more similar to those reported in the United States and Europe. It is possible to explain this difference in eradication rates in a number of ways. The first is the difference in *CYP 2C19*genetic polymorphism rates between the two countries. A higher prevalence of CYP 2C19 poor metabolizers was found in Japan^52,53^ as a result Japanese population will show greater efficacy of STT by boosting the acid inhibitory effect of PPIs. *CYP 3A4/5*genotypes are another genetic variation that could influence eradication rates. The second factor to consider is the difference in BMI between the two populations. The majority of Japanese study participants had a BMI below 25. However, in our study, the median BMI was found to be 27.39. Lower eradication rates were achieved in patients with larger BMI receiving vonoprazan dual therapy. This could be attributed to the lower amoxicillin plasma concentrations in these patients due to altered pharmacokinetic parameters including protein binding, distribution, metabolism, and elimination. Increasing adipose tissue, for instance, increases the volume of distribution of antibiotics, such as amoxicillin^54^. Thirdly, the prevailing local resistance patterns to antimicrobials between the two different geographical areas will certainly have an impact on the eradication rates of the regimens. Higher resistance rates to both amoxicillin and clarithromycin were noticed in Egypt^55–57^. Another potential explanation for the difference in eradication rates could be the stronger inhibition of gastric acid secretion in the Japanese population. Vonoprazan achieved a higher intragastric pH > 4 holding time ratio (HTR) in the Japanese population than in the UK population^58^. This may be the case in the Egyptian population as well, although more research is needed to confirm this hypothesis. Lastly, the difference in compliance rates to medications between the reviewed Japanese studies that reported compliance rates exceeding 90%^26,34^ and the present study may have contributed to differences in treatment outcomes as well. In the current study 71.87%, 70.96% and 69.69% of subjects receiving STT, VDT and VTT regimens, respectively, completed more than 90% of their medications. The median compliance rate was 100% in both the STT and the VTT groups which are administered twice daily, and 97.14% in the VDT group which is administered three times daily. In other words, higher dosing frequency translates into reduced compliance with treatment regimens.

Comparable tolerability and safety profiles were found across treatment groups. In terms of adverse event rates, no significant differences were observed between the groups apart from taste disturbance, which was the lowest in the VDT group. The VDT group also had the lowest number of patients experiencing severe adverse events leading to discontinuation of treatment as well as the lowest number of adverse events per subject. Noteworthy, adverse events especially severe ones would definitely impact the regimen’s feasibility, practicality and acceptability. Striking a balance between efficacy, safety, and patient tolerance is critical for ensuring the regimen’s success in real-world clinical practice.

Regarding factors influencing *H. pylori *infection symptoms severity, females had higher symptom severity scores than males. This may be due to the fact that women tend to report more frequent, and more intense somatic symptoms than men^59^. Despite this, Basir et al. did not find a statistically significant association between *H. pylori *infection intensity and gender^60^. Additionally, patients who had not previously received the COVID-19 vaccination reported less severe gastrointestinal symptoms. However, this finding should be interpreted with caution due to the lack of a consistent trend that prevents drawing a clear conclusion on the effect of vaccination on symptom severity. A review of the literature revealed a potential link between long-term gastrointestinal symptoms and the COVID-19 vaccination^61,62^. As well as this, a weak direct correlation was observed between patients’ BMI and their symptom severity scores. Foster et al. found that morbidly obese patients experienced more intense gastrointestinal symptoms^63^. Obesity has also been linked to non-specific colitis and GERD^64,65^, which in turn aggravates *H. pylori’s* gastrointestinal symptoms severity.

Finally, it is imperative that we take into account the limitations of this study. First, it is an open-label, single-center study which lacks a placebo-controlled group. This could limit the generalizability and depth of the study findings as well as constrain the generalization of the findings to other geographical regions. Second, the detection of *H. pylori* infection was carried out qualitatively by stool antigen test, rather than quantitatively, which does not allow detection of the effect of pretreatment bacterial load on *H. pylori* eradication and monitoring of *H. pylori* levels after treatment. Third, factors related to treatment success or failure were not addressed in this study. For example, antibiotic susceptibility testing, *CYP 2C19 or CYP 3A4* genotyping, and 24-hour intragastric pH measurement were not conducted. Finally, high loss to follow-up rate although addressed statistically through conducting sensitivity analysis, sensitivity analysis results should be framed more cautiously.

## Conclusion

This RCT provides the first assessment of the efficacy of vonoprazan-based regimens versus the widely used standard triple regimen in the Egyptian population. The study findings showed suboptimal cure rates of the three treatment regimens emphasizing the need for optimizing the dosing and frequency of existing treatment regimens and encouraging further research on developing new regimens using new antimicrobial agents in order to improve eradication rates that are currently in decline. VDT demonstrated comparable eradication rates with fewer adverse effects, prolonged gastric acid inhibition, a reduced effect of *CYP 2C19* polymorphism, reduced cost and with the use of a single antimicrobial agent. Accordingly, this regimen can be a valuable alternative to conventional PPI-based treatment regimens which usually involve two or three partly unnecessary antibiotics resulting in antibiotic overuse that leads to the development of antimicrobial resistance. The present study should be followed up by further multicenter studies involving a large number of participants to verify its findings and include cost-effectiveness analyses of vonoprazan-based regimens and alternative treatment options for *H. pylori* infection.

## Data Availability

All data generated or analyzed during this study are included in this published article.
